# Unveiling the Potential of Single‐Cell Encapsulation in Biomedical Applications: Current Advances and Future Perspectives

**DOI:** 10.1002/smsc.202300332

**Published:** 2024-03-25

**Authors:** Manuel Pires‐Santos, Sara Nadine, João F. Mano

**Affiliations:** ^1^ CICECO ‐ Aveiro Institute of Materials Department of Chemistry University of Aveiro Campus Universitário de Santiago 3810‐193 Aveiro Portugal

**Keywords:** cell analysis, cell therapy, microgels, Poisson distribution, single‐cell encapsulation, sorting strategies

## Abstract

The encapsulation of single cells has emerged as a promising field in recent years, owing to its potential applications in cell‐based therapeutics, bioprinting, in vitro cell culture, high‐throughput screening, and diagnostics. Single‐cell units offer several advantages, including compatibility with standard imaging techniques, superior diffusion rates, and lower material‐to‐cell volume ratios. They also serve as effective carriers for targeted drug delivery, allowing precise administration of therapeutics in cell‐mediated quantities. Moreover, single‐cell units exhibit improved circulation potential throughout the vasculature, with a reduced likelihood of entrapment compared to multicell strategies. However, the production of single‐cell units from random dispersion of cells follows the Poisson distribution, requiring the separation of empty and multicell units from single‐cell ones. Various methods have been developed to address this challenge; nevertheless, the majority of these strategies are either expensive or time‐consuming. This review provides an in‐depth analysis of the advantages and limitations of single‐cell units and their applications, as well as a comprehensive overview of the most used techniques for single‐cell encapsulation and sorting strategies.

## Graphical abstract

Single‐cell encapsulation approaches such as droplets, microgels and shells, offer precise control over thecell microenvironment. While droplets excel in short‐term analyses, hydrogel and nanoencapsulation strategies offer versatility, particularly in tissue engineering. This review presents a comprehensive investigation of techniques, advantages, and applications while also delving into essential sorting strategies aligned with the Poisson distribution.

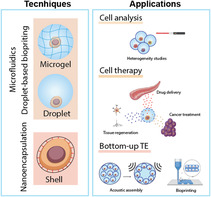

## 1 Introduction

Cell encapsulation has emerged as a shielding technique, protecting transplanted cells from the host's immune system response, while promoting long‐term cell viability and function.^[^ ^1^ ^]^ In 1933, Vincenzo Bisceglie encapsulated tumor cells and implanted the capsules in a pig's abdominal cavity, which is regarded as the beginning of encapsulation technology.^[^ ^2^ ^]^ Later, Thomas Chang explored the idea of using semipermeable microcapsules for drug delivery.^[^ ^3^ ^]^ Preclinical trials have shown that implanting microencapsulated pancreatic islets in diabetic rats allowed for the maintenance of normoglycemia for almost 3 weeks, which is significantly longer than the survival time of the nonencapsulated islets group, which only survived between 6 and 8 days. Diabetes‐related symptoms, including polyuria and polydipsia, experienced a notable reduction.^[^ ^4^ ^]^ Across time, a plethora of approaches have been employed within the spectrum of cell encapsulation strategies. These methodologies have not only encompassed various health conditions but have also been extended to diverse biomedical applications. Currently, cell encapsulation strategies are being employed as biosensors, tissue regenerative agents, or even as containers for manufacturing biomolecules for pharmaceutical purposes. However, most of the reported technologies are focused on multicell encapsulation due to its ability to create highly complex and functional cellular systems, closely mimic the natural tissue environment, and meet cell quantity requirements for numerous purposes. Although less explored, single‐cell encapsulation holds significant importance, mainly for studying cell behavior at the individual level.

Single‐cell encapsulation is emerging as a relevant topic in science, particularly due to its advantages, such as its small size and precise control over each cell's microenvironment. Several approaches to individualize cells have been explored, with microfluidics‐based methodologies, being the most prevalent. Nevertheless, independently of the technique employed, we consider that there are three types of encapsulations of single cells based on the suspension environment cells that are embedded: droplets, microgels, or shells. Taking this into consideration, a categorization into three classes was made: “droplet‐based encapsulation,” “hydrogel‐based encapsulation,” and “nanoencapsulation.” All of them present different characteristics and features for specific applications. Droplet‐based encapsulation encompasses diverse droplet‐forming techniques wherein cells are suspended in a liquid solution, devoid of any cross‐linking agents. The hydrogel‐based encapsulation centers on the generation of viscous core or cross‐linked systems (such as microgels) from a cell–polymer solution. Finally, nanoencapsulation involves any strategy where a nanosized and protective shell is generated around the cell (capsule), achieved through a range of cell–polymer interactions. Overall, the current work on single‐cell droplets is very focused on short‐term cell analysis (antibiotic suitability testing, heterogeneity studies, cancer research, among others), whereas hydrogel‐based encapsulation and nanoencapsulation have been proven to be more versatile approaches, offering broader applications particularly within the tissue engineering field.

Overall, this review brings a new insight into the different single‐cell encapsulation strategies and categorizes them based on the type of encapsulation employed. The review provides a comprehensive overview, ranging from conventional to recent techniques, detailing specific advantages and disadvantages, as well as current and prospective applications. It also encompasses the main sorting strategies, essential for encapsulation procedures following the Poisson distribution.

## 2 Single‐Cell Encapsulation: Advantages, Disadvantages, and Applications

Single‐cell units are usually characterized by a container that encapsulates a single cell. The encapsulation is often achieved by compartmentalizing the cell within droplets and hydrogels, utilizing solutions at volumes ranging from picolitres to microliters. Alternatively, the cell may be enveloped by protective shells.

A key benefit of employing single‐cell encapsulation is the ability to investigate cells at the individual level. Exploring cellular heterogeneity at the individual level emerges as a potent and transformative strategy in modern biological research. By meticulously analyzing cells individually, this approach reveals new information about cell biology that often goes unnoticed in broader aggregate analyses. This heightened resolution not only exposes these differences but also facilitates the identification of rare cellular subpopulations with key roles in disease initiation, progression, and treatment responses. As a result, our understanding of the intrinsic molecular and functional mechanisms controlling cellular dynamics deepens, enabling a more precise and comprehensive scheme of the complexity underlying biological systems. As molecules released from individual cells may be present at low concentrations, population‐based studies will not identify any signals from the minority of aberrant cells, consequently leading to the loss of crucial insights. In contrast, the exploration of cells at the individual level empowers the acquisition of meticulous and comprehensive insights into the cells’ physiology, enriching our understanding in unprecedented ways and underscoring the significance of this personalized vantage point in unraveling the complexities of biological systems.^[^ ^5^, ^6^ ^]^

Besides the advantage of assessing cellular heterogeneity, single‐cell encapsulation is an interesting technology due mainly to its small size. For instance, this strategy demonstrates direct compatibility with common imaging methods like confocal microscopy, where the size of the sample eliminates the need to section it first. Additionally, it offers better diffusion rates and lower material‐to‐cell volume ratios and allows cells to create 3D high‐resolution microniches that permit excellent control over the culture, mainly when cells are incorporated within cross‐linked polymers. This is visualized in the study conducted by Chicheportiche and Reach, where rat islets of Langerhans were encapsulated into two distinct‐sized capsules, namely, large (650 μm) and small (350 μm). The capsules were formed by the interaction of alginate with polylysine. Cells encapsulated in smaller microcapsules displayed enhanced insulin release when compared to cells in larger microcapsules.^[^ ^7^ ^]^ Single‐cell units can also be advantageous for in vivo applications because they can be adjusted or fitted into smaller spaces, allowing the control of the biomaterial's location after administration or injection.^[^ ^8^ ^]^ This technology also offers precise microscale control in the assembly of complex tissues and enables the delivery of cells via different routes of administration.^[^ ^9^ ^]^ All these features facilitate pharmacological studies and regenerative medicine applications.^[^ ^7^, ^8^, ^10^, ^11^ ^]^

However, the single‐cell encapsulation approach presents some disadvantages.^[^ ^8^ ^]^ One of the limitations is related to the size of single‐cell units when implanted, as different sizes may influence the host's immune response.^[^ ^12^ ^]^

Additionally, another drawback is related to the actual yield of the current techniques used to produce single‐cell‐laden units, mainly when utilizing a cell dispersion method. The number of encapsulated cells in each unit naturally follows the Poisson distribution (1), where *k* is the total number of cells present in the droplet and *λ* is the average cell count per droplet, which is altered by adjusting the cell density. Thus, a maximum of 37% of them can only contain one cell, and the rest of the units contain either no cells or multiple cells, leading to a small number of single‐cell units.^[^ ^13^, ^14^ ^]^

(1) $$P\left(\lambda ;k\right)\;=\lambda^{k}\;\frac{e^{-\lambda }}{k!}$$

The main challenge is to surpass the Poisson distribution to increase encapsulation efficiency; here, we showcase some studies where researchers were able to move beyond the limits set by the Poisson distribution, by applying new cell organization technologies. Andreas Link et al. used an active encapsulation method by applying an external acoustic field to encapsulate individual red blood cells into droplets in a single step. The yield of encapsulation attained 98% of single‐cell droplets while also maintaining high encapsulation rates.^[^ ^15^ ^]^ The bead‐ordered arrangement droplet (BOAD) system was suggested as a novel approach to creating single‐cell units.^[^ ^14^ ^]^ The BOAD system deftly blends sheath flow, Dean vortex, and compression flow channels to create an orderly organization of particles, increasing the single‐bead encapsulation efficiency to a maximum of 86%. Additionally, a different study shows that the combination of viscoelastic properties and droplet formation in microfluidic devices can overcome the Poisson limit. The researchers observed controlled single‐cell encapsulation with higher efficiency than the Poisson limit for all the viscoelastic fluids tested. They also achieved controlled coencapsulation of particles with efficiencies up to two‐fold larger than the Poisson limit.^[^ ^16^ ^]^ Nevertheless, when the encapsulation procedures follow the conventional Poisson distribution, it is necessary to sort the single‐cell units from those that are empty or contain multiple cells. This topic will be further explored and discussed later in Section 4.

**Figure 1:**
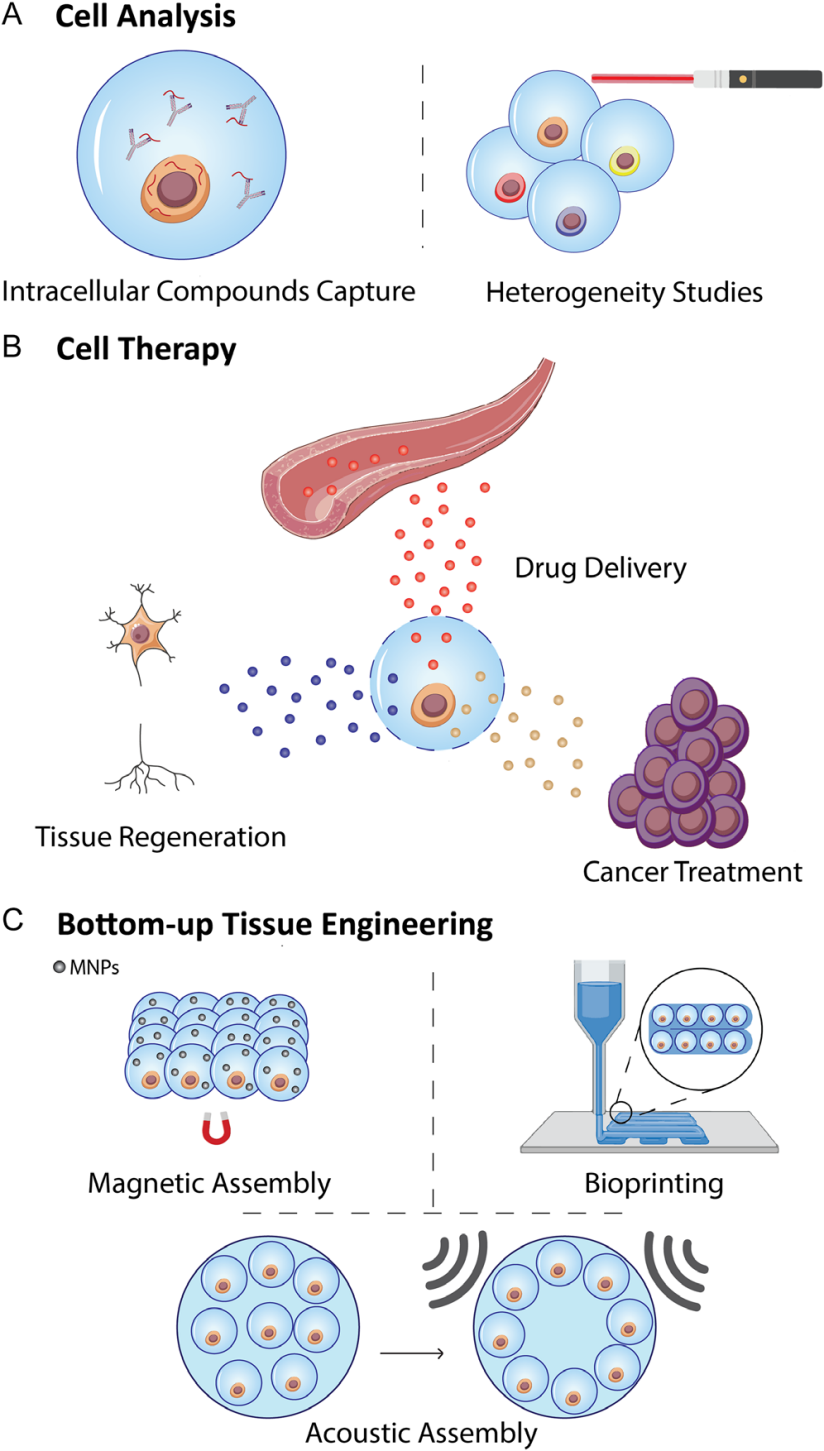
Applications of single‐cell strategies. A) Cell analysis: examples include antibody trapping or studying the stem cell heterogeneity inside a population. B) Cell therapy: illustrated by cytokines release to enhance the immune system response or tissue regeneration. C) Tissue engineering strategies: single‐cell units can be utilized as building blocks for bottom‐up strategies.

This technique finds utility in various areas, such as the control of cell fate and screening, in situ capturing of intracellular compounds, cell therapy, and modular tissue engineering (**Figure** **1**).

### 2.1 Cell Analysis

Within the cell analysis field (Figure 1A), there is a diversity of tools, such as small molecule detection, genomics, transcriptomics, and proteomics, that can be used to evaluate cells at a single level. One of the objectives is to analyze specific changes at the individual cell level, prompted by a precise stimulus. Moreover, this technique extends to the generation of specific intracellular compounds, including but not limited to antibodies.^[^ ^17^, ^18^ ^]^ The work developed by Loveday and co‐workers on Influenza Virus A is a great illustration of investigating cellular responses to a stimulus. Different adherent cell lines (alveolar basal epithelial cells (A549), Madin‐Darby Canine Kidney (MDCK) cells, and *siat7e* cells) were encapsulated individually in droplets and cocultured with the virus to promote infection and propagate enveloped viruses. This strategy proved to be efficient for Influenza Virus A propagation, evidencing that adherent single cells can be utilized for studying viral infections in liquid microenvironments and paving the way for prospective high‐throughput analysis of individual host cell interactions within confined microenvironments throughout the viral life cycle.^[^ ^19^ ^]^ Pursuing a different aim, liver cancer cell subpopulations (HepG2, HCCLM3, and MHCC97) and a normal liver cell line (HHL‐5) were encapsulated individually in microgels. The encapsulation aimed to selectively retain specific cancer‐related microRNAs (miRNA‐21, 122, and 22) for targeted investigation. The purpose of the work was to develop a standardized methodology to target miRNAs with discriminatory potential, distinguishing between liver cancer cell lines and their normal counterparts. To achieve this, single cells were encapsulated in polyethylene glycol (PEG) hydrogel precursor functionalized with miRNA capture probes using a microfluidic device. After cell cytolysis, results indicated that microgels showed a good miRNA retention rate and demonstrated a pattern to distinguish each subpopulation of liver cell lines.^[^ ^20^ ^]^ For instance, the heterogeneity among cytotoxic T cells (CTLs) can also be analyzed utilizing a single‐cell‐based method. It is known that naïve CTLs, when activated by antigen‐presenting cells, can differentiate into effector CTLs. The effector CTLs exhibit multiple subpopulations, being distinguished by the type of cytokines released.^[^ ^21^ ^]^ An innovative multiparameter single‐cell analysis was proposed for decoding the CTL activation profile using a droplet‐based microfluidic platform. In this method, individual CTLs were encapsulated within ultralow melting point agarose microgels, enabling the detection and identification of different subpopulations based on interferon‐gamma (IFN‐γ), tumor necrosis factor‐alpha (TNF‐α), and interleukin‐2 (IL‐2) secretion profiles, alongside surface marker expressions like CD69 and CD25. The high‐throughput analysis was carried out by flow cytometry, with the added advantage of sorting when required.^[^ ^22^ ^]^

Analyzing cells at a single‐cell level offers a more comprehensive understanding of the physiological conditions and functions of diverse cell populations.^[^ ^23^ ^]^ Depending on the technology applied, there is the possibility of high‐throughput single‐cell screening of millions of cells.^[^ ^24^, ^25^ ^]^ This analytical approach allows to explore various aspects of a certain cell population, including cell size, gene expression profiles, and growth features.^[^ ^26^, ^27^, ^28^ ^]^ Single‐cell encapsulation enables researchers to study the human proteome^[^ ^29^ ^]^ and facilitates the generation of draft genomes of several species, even encompassing complex ecosystems like the gut microbiome.^[^ ^30^ ^]^

### 2.2 Cell Therapy

Cell therapy involves the transplantation of human viable cells and is a promising tool for several therapeutic areas, including regenerative medicine, immunotherapy, and cancer therapy.^[^ ^31^ ^]^ Essentially, cell therapy aims to restore the function of damaged cells or tissues by delivering either stem or somatic cells. Within the stem/stromal cell category, multipotent stromal cells such as mesenchymal‐derived stromal cells (MSCs), hematopoietic stem cells (HSCs), and neural stem cells (NSCs) are the most prevalent types under clinical investigation.^[^ ^32^ ^]^

MSCs exhibit a potential to differentiate into different phenotypes, including osteoblasts, adipocytes, hepatocytes, cardiac and skeletal muscle cells, as well as neural cells, among others,^[^ ^33^, ^34^, ^35^ ^]^ and for that reason, they are used in various applications. Nevertheless, in the single‐cell field, MSCs have been used mainly to differentiate toward the osteogenic lineage. Zehao Pan successfully enclosed individual MSCs within alginate microgels and induced their differentiation into osteoblasts, maintaining cell viability for a minimum of 7 days. Furthermore, these single‐cell‐laden microgels exhibited versatility as modular bioink units, making them suitable for diverse 3D printing applications. We will explore this aspect in the following Subsection 2.3.^[^ ^36^ ^]^ On the other hand, pluripotent stem cells, including embryonic stem cells (ESCs) and induced pluripotent stem cells (iPSCs), hold an unused potential as sources for cell therapy. However, these sources are relatively less explored due to ethical considerations, tumorigenic tendencies, immunological reactions, and inherent variability.^[^ ^31^, ^37^, ^38^ ^]^

Besides stromal cells, immune cells such as T cells, dendritic cells, and CAR‐T cells have been widely employed in cancer cell‐based therapies. Dendritic cell vaccines are being proposed to increase the tumor antigen presentation and consequently boost the immune system response. Simultaneously, T cell therapy is based on the direct recognition of tumor antigens by the infused lymphocytes, leading to effective tumor elimination.^[^ ^39^ ^]^ Nevertheless, cell therapy's main problem is related to the poor engraftment capacity of implanted cells and immune rejection. To overcome these issues, the encapsulation of therapeutic single cells is being proposed as a shielding strategy (Figure 1B). Recently, a single‐cell‐laden gel droplet was employed as a noninvasive T cell adoptive therapy to treat solid tumors. Briefly, T cells isolated from the blood of tumor‐bearing mice were encapsulated in alginate microgels using a microfluidic chip. The encapsulation of cells was combined with adjuvants (Toll‐like receptor 1/2 agonist (TLR1/2) and interleukin‐21 (IL‐21)) to enhance proliferation and reduce differentiation. The microenvironment of single‐cell microgels enabled faster cell proliferation while maintaining differentiation at lower levels compared to traditional culture flasks. After CD8^+^ PD‐1^+^ cell proliferation, gel droplets containing cells, cytokines, and adjuvants were subcutaneously injected into the melanoma mice, promoting a robust immune response toward tumor cells, and increasing the global survival rate.^[^ ^40^ ^]^ Fang and co‐workers developed an innovative single‐cell encapsulation technology for pinpointing T cell candidates with optimal properties for therapeutic application. This technology relied on the real‐time monitoring of individual T cell units through a photodetachable DNA‐copolymer nanocage on the cell membrane. Upon T cells secreting IFN‐γ, a marker of CTL activation, the DNA‐copolymer nanocage encapsulating a self‐quenched IFN‐γ recognition aptamer emitted a fluorescence signal which allowed to distinguish and select the appropriate cells through a fluorescence cell sorting technique, such as flow cytometry. Following sorting, the exposure for 5 min to UV light detached the nanocage without compromising T cell viability, activation, and antitumoral efficacy.^[^ ^41^ ^]^

The almost untouched potential of single‐cell therapy lies in its focused approach, dealing with a limited number of cells rather than millions at once. This targeted strategy reduces immunogenicity and minimizes the probability of adverse effects. The use of small encapsulation units not only allows for safer administration routes, such as intravenous delivery without entrapment risks but also opens doors to innovative therapeutic applications. Understanding single‐cell therapy means improving methods and appreciating the benefits of dealing with fewer cells. This helps create more accurate and safer ways to approach regenerative medicine and treatments.

### 2.3 Bottom‐Up Tissue Engineering

Within the tissue engineering field, a significant surge in the investigation of single‐cell units has emerged, unveiling promising avenues for the advancement of bottom‐up methodologies (Figure 1C). There is a plethora of techniques that are able to organize cell units, such as bioprinting,^[^ ^10^ ^]^ magnetic assembly,^[^ ^42^, ^43^ ^]^ and acoustic assembly.^[^ ^43^, ^44^ ^]^ A notable example of this strategy comes from the work of Sha et al. who harnessed microfluidics to develop a single‐cell approach for bone tissue engineering. In more detail, single rat MSCs were encapsulated in alginate microgels, which were prepared with either calcium complexes of calcium–ethylenediaminetetraacetic acid (Ca–EDTA) or calcium–nitrilotriacetic (Ca–NTA). As the Ca–NTA showed better cell viability and more metabolically active cells, it was chosen to study the osteogenic potential. Results indicated that encapsulated MSCs were able to undergo osteogenic differentiation, initiating the mineralization process within the microgel matrix.^[^ ^45^ ^]^ Single‐cell units can also be employed as building blocks for the concept of bottom‐up tissue engineering. Polyethylene glycol diacrylate (PEGDA) microgels (35 μm in diameter) containing single MSCs or bovine chondrocytes were employed as building blocks to create a modular bioink. These single‐cell‐laden microgels generated by a microfluidic device and sorted by flow cytometry were incorporated in different bioinks, including PEGDA, dextran‐tyramine, collagen, alginate/gelatin methacryloyl (GelMA), and alginate. The macrostructures were then developed by various techniques, such as photolithography, emulsification, injection molding, 3D printing, and wet spinning. The obtained results highlight the versatility, straightforwardness, and cost‐effectiveness of the generated single‐cell microgels, enabling the creation of multiple modular bioinks. Furthermore, it evidences that single‐cell microgels can be incorporated into a diverse array of injectable macrostructures through varied biofabrication technologies.^[^ ^10^ ^]^

Finally, magnetic and acoustic assembly remain relatively unexplored in the single‐cell encapsulation field, despite being well‐established in multicell approaches. Both techniques offer rapid and straightforward assembly processes, leading to a plethora of final constructs. Magnetism enables the formation of stratified cell structures, such as cell sheets^[^ ^42^, ^46^ ^]^ or spheroids^[^ ^47^ ^]^ (2D or 3D cell culture), within a matter of seconds. This ability allows the formation of complex cell constructs which better mimic the in vivo environment. In the case of acoustic assembly, it not only facilitates the assembly of basic constructs like spheroids but also enables the creation of complex 3D structures with various types of cells and spatial arrangements.^[^ ^48^, ^49^, ^50^ ^]^ Acoustic waves are able to give rise to a variety of cell‐organized patterns within seconds to minutes. Although the referenced examples of the literature do not utilize single‐cell units, they demonstrate the immense potential of combining single‐cell strategies with different assembly techniques. Combining the major advantages of single‐cell units, namely, the individual cell‐to‐cell control, with different assembly techniques, opens the possibility of constructing cell scaffolds with higher complexity, where is possible to control the fate and the alignment of each cell. Similar to single‐cell therapy, the advantages stand from using fewer cells and smaller encapsulation units. This helps minimize side effects and immunogenicity, improves administration routes, and enables precise control over the microenvironment of each encapsulated cell. This precision allows for the creation of diverse and complex tissues with accurate placement of cells, a task that is more challenging in conventional encapsulation approaches.

## 3 Single‐Cell Encapsulation Technologies and Applications

The range of applications can vary depending on the methodologies employed to develop single‐cell units. Our categorization is based on the suspension environment cells inhabit, giving rise to three distinct classes: “droplet‐based encapsulation,” “hydrogel‐based encapsulation,” and “nanoencapsulation.” Droplet‐based encapsulation encompasses diverse droplet‐forming techniques wherein cells are suspended in a liquid solution, devoid of any cross‐linking agents, like cell culture medium or phosphate‐buffered saline (PBS). The hydrogel‐based encapsulation centers on the generation of viscous core or cross‐linked systems (such as microgels) from a cell–polymer solution. On the other hand, nanoencapsulation involves any strategy where a nanosized and protective shell is generated around the cell (capsule), achieved through a range of cell–polymer interactions, including electrochemical bonds and affinity reactions. It is worth noting that these three techniques have the potential to coexist within a singular approach.

### 3.1 Droplet‐Based Encapsulation

**Figure 2:**
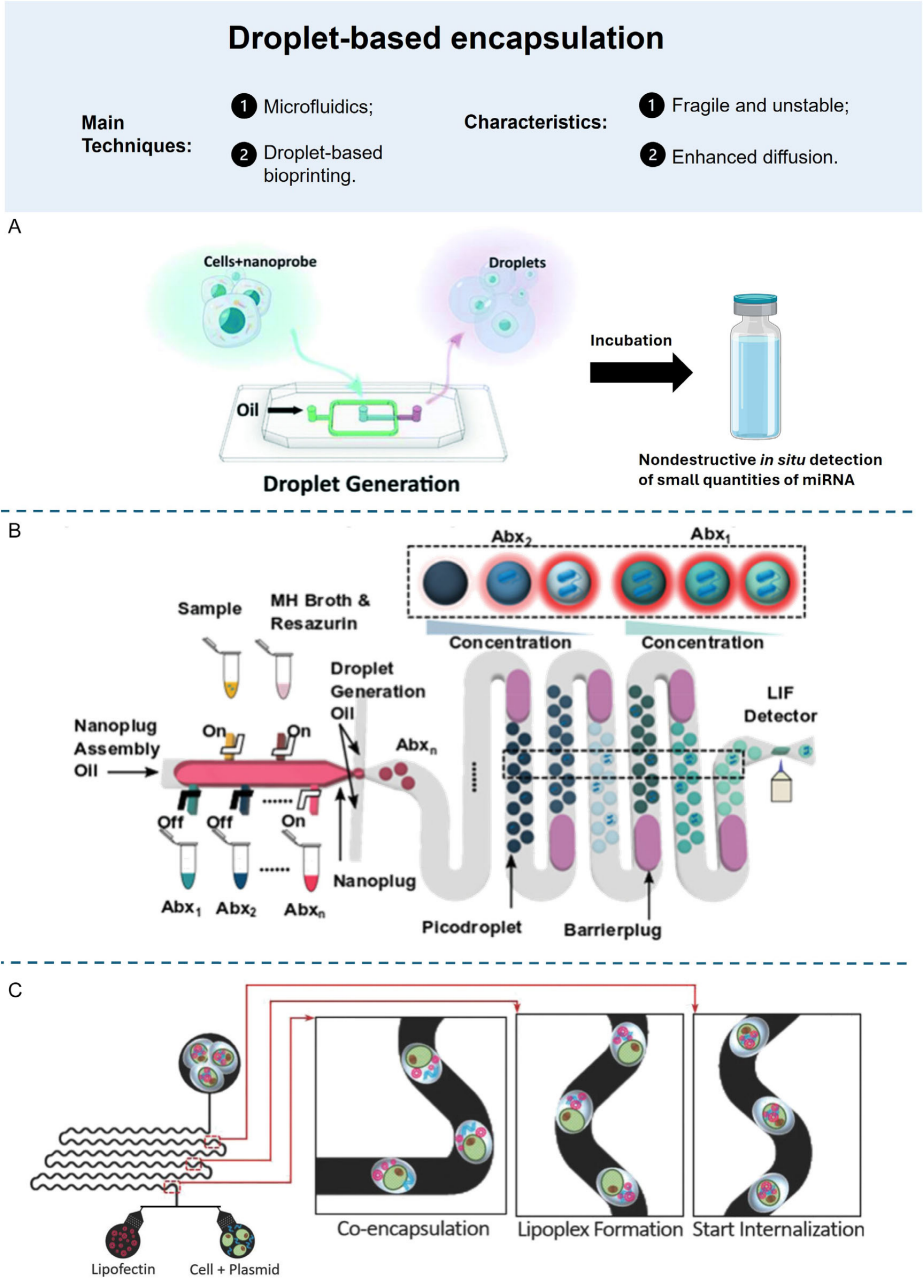
Main production techniques and characteristics of droplet‐based encapsulation. A) A traditional droplet‐based microfluidic was upgraded with imaging technology, facilitating nondestructive in situ detection of small quantities of miRNA, for single‐cell analysis. Reproduced with permission.^[^ ^60^ ^]^ Copyright 2022, The Royal Society of Chemistry. B) A new method for antibiotics’ susceptibility testing using droplet microfluidics was fabricated, with the ability to test 32 different types of antibiotics within the same machine. Reproduced with permission.^[^ ^63^ ^]^ Copyright 2021, Wiley‐VCH GmbH. C) Single‐cell droplets were applied for gene therapy studies, specifically, lipofection. Importantly, this method significantly enhanced the plasmid's delivery efficiency from around 5% to ≈50% across diverse suspension cell lines.

Microfluidics is considered a highly powerful technique to fabricate spherical droplets, particularly those with a very small size (<100 μm), finding extensive application in single‐cell encapsulation (**Figure** **2**).^[^ ^10^, ^51^ ^]^ The conventional approach involves injecting highly diluted cell solutions into microfluidic chips to generate microdroplets, each one containing just one cell.^[^ ^52^ ^]^ Despite its widespread adoption, this technique has some drawbacks, mainly associated with the use of oil and surfactants due to their cytotoxicity and the challenges associated with their removal.^[^ ^53^ ^]^ The majority of the single‐cell droplet research uses microfluidic as the main technique; however, droplets can be formed through other methodologies, such as acoustic systems,^[^ ^15^, ^54^ ^]^ jet printing,^[^ ^53^ ^]^ or even cell trapping by integrated dielectrophoresis.^[^ ^11^ ^]^ Independently of the technique employed, the research using droplets is centered on the analysis and study of stem cell fate,^[^ ^55^ ^]^ cell heterogeneity,^[^ ^56^ ^]^ cell lines proliferation,^[^ ^57^ ^]^ and RNA^[^ ^58^ ^]^ and DNA^[^ ^59^ ^]^ evaluation. **Table** **1** provides a comprehensive summary of techniques employed for the creation of single‐cell units via droplet‐based encapsulation, along with their corresponding cell type, culture conditions, and applications.

**Table 1:** Overview of works involving droplet‐based single‐cell encapsulation. DMEM (Dulbecco's Modified Eagle Medium); PBS (phosphate buffer saline); IMDM (Iscove's Modified Dulbecco's Medium); *E. coli* (*Escherichia coli*); RBC (red blood cell); *S. cerevisiae* (*Saccharomyces cerevisiae*); dH_2_O (distilled water); MDCK (Madin–Darby canine kidney); *S. aureus* (*Staphylococcus aureus*); *S. venezuelae* (*Streptomyces venezuelae*); TAP (TRIS acetate phosphate); YDP (yeast extract peptone dextrose); FBS (fetal bovine serum); LB (Lysogeny broth); *B. licheniformis* (*Bacillus licheniformis*); *B. subtilis* (*Bacillus subtilis*); MH (Mueller Hinton); α‐MEM (minimum essential medium alfa); HUVECs (human umbilical vein endothelial cells); ddH_2_O (double distilled water); *P. rhodozyma* (*Phaffia rhodozyma*); BMMC (human bone marrow mononuclear cells); PBMC (peripheral blood mononuclear cell); *S. pneumoniae* (*Streptococcus pneumoniae*); IPS (induced pluripotent stem cell); and HSC (hematopoietic stem cells)

Author	Technique	Solvent	Cell type	Application
Zhiliang Bai et al.^[^ ^58^ ^]^	Integrated dielectrophoresis trapping	DMEM medium	NIH3T3 and HEK293	Cell analysis
Yacine Bounab et al.^[^ ^121^ ^]^	Emulsification (water–oil)	RPMI‐1640	T cell, B cell, and monocytes	Cell analysis
Eric Brouzes et al.^[^ ^61^ ^]^	Emulsification (water–oil)	RPMI‐1640	U937	Cell analysis
Kara K. Brower et al.^[^ ^114^ ^]^	Double emulsion (water−oil−water)	PBS and OptiPrep	mESC	Cell analysis
Huichao Chai et al.^[^ ^115^ ^]^	Emulsification (water–oil)	PBS and Pluronic F‐12	MCF‐7	Cell analysis
Keke Chen et al.^[^ ^54^ ^]^	Acoustic system	RPMI‐1640	4T1	Cell analysis
Aaron Chen et al.^[^ ^71^ ^]^	Emulsification (water–oil)	PBS	BT‐474 and RBC	Sorting platform
Meng Ting Chung et al.^[^ ^72^ ^]^	Emulsification (water–oil)	PBS and OptiPrep	Jurkat, K562, and Neuro‐2A	Sorting platform
Joseph de Rutte et al.^[^ ^73^ ^]^	Emulsification (water–oil)	DMEM, IMDM and RPMI‐1640	CHO DP‐12, ExpiCHO, HEK293, HyHel‐5 and 9E10	Sorting platform
Bachir El Debs et al.^[^ ^122^ ^]^	Emulsification (water–oil)	RPMI‐1640	4E3 and Elec‐403	Cell analysis
Jon F. Edd et al.^[^ ^123^ ^]^	Emulsification (water–oil)	PBS	HL60	Cell analysis
Jack Harrington et al.^[^ ^124^ ^]^	Emulsification (water–oil)	PBS	HEK29 and THP‐1	Cell analysis
Heon‐Ho Jeong et al.^[^ ^125^ ^]^	Emulsification (water–oil)	PBS	*E. coli*	Clinical analysis
Tengyang Jing et al.^[^ ^116^ ^]^	Emulsification (water–oil)	RPMI‐1640	PC‐9	Sorting platform
Younggeun Jo et al.^[^ ^118^ ^]^	Emulsification (water–oil)	f/2 medium	*Thalassiosira eccentrica*	Sorting platform
Gopakumar Kamalakshakurup et al.^[^ ^117^ ^]^	Emulsification (water–oil)	dH_2_O, lipids, glycerols and surfactant	K‐562	Encapsulation efficiency
Mathias Girault et al.^[^ ^120^ ^]^	Emulsification (water–oil)	f/2 medium	*Dunaliella tertiolecta* and *Phaeodactylum tricornutum*	Sorting platform
Evelien W. M. Kemna et al.^[^ ^13^ ^]^	Emulsification (water–oil) using Dean force	RPMI‐1640	HL60 and K562	Encapsulation efficiency
Mohammad Ali Khorshidi et al.^[^ ^126^ ^]^	Emulsification (water–oil)	HEK 293 T	HEK293	Cell analysis
Hyun Soo Kim et al.^[^ ^64^ ^]^	Emulsification (water–oil)	TAP and Chu 13	*Chlamydomonas reinhardtii* and *Botryococcus brauni*	Microalgal growth and oil production analysis
Luoquan Li et al.^[^ ^127^ ^]^	Emulsification (water–oil)	PBS	hHEK293T and mNIH3T3	Cell analysis
Xuan Li et al.^[^ ^65^ ^]^	Emulsification (water–oil)	Opti‐MEM	K562, THP‐1 and Jurkat	Gene therapy
Andreas Link et al.^[^ ^15^ ^]^	Emulsification (water–oil) + acoustic system	OptiPrep	RBC	Overcoming Poisson distribution
Hangrui Liu et al.^[^ ^62^ ^]^	Emulsification (water–oil)	YDP	*S. cerevisiae*	Cell analysis
Yang Liu et al.^[^ ^128^ ^]^	Emulsification (water–oil)	DMEM or RPMI‐1640	T2, Jurkat, NIH/3T3 (CRL‐1658), and 293 T	Cell analysis
Hangrui Liu et al.^[^ ^129^ ^]^	Emulsification (water–oil)	RPMI‐1640 and OptiPrep	THP‐1	Cell analysis
Emma Kate Loveday et al.^[^ ^19^ ^]^	Emulsification (water–oil)	Hams F‐12 or DMEM	A549 and MDCK	Study of virus life cycle
Ning Ma^[^ ^130^ ^]^	Emulsification (water–oil)	RPMI 164	K562 and Jurkat	Cell analysis
Amy Mongersun^[^ ^131^ ^]^	Emulsification (water–oil)	DMEM	U87 and K562	Cell analysis
Yuta Nakagawa et al.^[^ ^132^ ^]^	Emulsification (water–oil)	YPD–sorbitol	*S. cerevisiae*	Cell analysis
Maryam Navi et al.^[^ ^69^ ^]^	Emulsification (water–water)	Dextran	MCF‐7	Sorting platform
Dan Sun et al.^[^ ^60^ ^]^	Emulsification (water–oil)	DMEM	LO2, HeLa, HepG2 and MCF‐7	Cell analysis
Leon P. Pybus et al.^[^ ^57^ ^]^	Cyto‐Mine instrument	Culture medium (animal component free)	Different cell lines	Cell encapsulation platform
Kenza Samlali et al.^[^ ^133^ ^]^	Emulsification (water–oil) + electrode system	PBS or DMEM without FBS	NCI‐H1299 and MCF‐7	Cell encapsulation platform
Aude I. Segaliny et al.^[^ ^70^ ^]^	Emulsification (water–water)	RPMI 1640	Jurkat E6.1 and K562	T cell therapy
Guoyun Sun et al.^[^ ^55^ ^]^	Emulsification (water–oil)	Williams E medium	HepaRG	Sorting platform
Dan Sun et al.^[^ ^56^ ^]^	Emulsification (water–water)	DMEM	HepG2	Cell analysis
Stanislav S. Terekhov et al.^[^ ^134^ ^]^	Emulsification (water–oil)	YDP or 2YT (depending on the cells)	*S. aureus, S. venezuelae and E. coli*	Cell analysis
Eujin Um et al.^[^ ^135^ ^]^	Emulsification (water–oil)	dH_2_O	*E. coli*	Storage of single‐cell droplets
Ming Wang et al.^[^ ^136^ ^]^	Emulsification (water–oil)	Gelatin microparticles + DMEM or MEGM	A549, MCF10A, MCF‐7 and MSAMB‐231	Cell analysis
Li Wu et al.^[^ ^53^ ^]^	Electrohydrodynamic jet printing + emulsification (water–water)	PBS or Isoton II	k562	Encapsulation and sorting platform
Tianze Xie et al.^[^ ^137^ ^]^	Emulsification (water–oil)	MEM	U‐87 MG	Cell analysis
Teng Xu et al.^[^ ^138^ ^]^	Emulsification (water–oil)	dH_2_O	*E. coli*	Cell analysis
Chun Guang Yang et al.^[^ ^139^ ^]^	Emulsification (water–oil)	Human whole blood	human blood cells	Cell encapsulation platform
Huiling Yuan et al.^[^ ^140^ ^]^	Emulsification (water–oil)	LB medium	*B. licheniformis and B. subtilis*	Cell analysis
Yuan Yuan et al.^[^ ^141^ ^]^	Emulsification (water–oil)	α‐MEM and DMEM (depending on the cells)	NK‐92 MI and K562	Cell analysis
Weifei Zhang et al.^[^ ^87^ ^]^	Emulsification (water–oil)	Serum‐free medium	HUVECs and A549	Cell analysis
Pengfei Zhang et al.^[^ ^63^ ^]^	Emulsification (water–oil)	MH culture broth	*E. coli*	Antibiotic susceptibility analysis
Qiang Zhang et al.^[^ ^142^ ^]^	Emulsification (water–oil)	ddH_2_O	*P. rhodozyma*	Cell analysis
Grace X.Y. Zheng et al.^[^ ^143^ ^]^	Emulsification (water–oil)	PBS	BMMC and PBMC	Cell analysis
Jianwei Zhong et al.^[^ ^119^ ^]^	Emulsification (water–oil)	BTXPRESS Low Conductivity Medium	MCF‐7	Sorting platform
Derek Thibault et al.^[^ ^59^ ^]^	Emulsification (water–oil)	Todd Hewitt Broth	*S. pneumoniae*	Cell analysis
Pengfei Zhang et al.^[^ ^144^ ^]^	Bioprinting	PBS	NIH 3T3, IPS and HSC	Organoid science, and cell therapies.
Iain C. Clark et al.^[^ ^145^ ^]^	Emulsification (water–oil)	PBS	HEK 293T and NIH 3T3	Cell analysis
Tao Tan et al.^[^ ^146^ ^]^	Emulsification (water–oil)	DMEM and RPMI 1640	MDA‐MB‐231 and MKN‐45	Encapsulation platform
Long Chen et al.^[^ ^147^ ^]^	Emulsification (water–oil)	PBS	HEK 293T	Encapsulation platform
Siyuan Zhuang et al.^[^ ^148^ ^]^	Emulsification (water–oil–water)	PBS	*S. cerevisiae*	Cell analysis

Distinct researchers have recently achieved remarkable advancements in generating single‐cell droplets. Recently, Dan Sun et al. developed an improved microfluidic droplet platform for single‐cell analysis (Figure 2A). The traditional droplet‐based microfluidic was upgraded with imaging technology, facilitating nondestructive in situ detection of small quantities of miRNA‐21 in a single living cell. The cells were first cultured with a nanoprobe, responsible for the miRNA‐21 detection, for 4 h of their complete internalization. Only after the cells were encapsulated in droplets and incubated for 4 h, allowing the binding between the complementary DNA present in the nanoprobe and miRNA‐21, and consequently its detection. The cells were suspended in Dulbecco's Modified Eagle Medium (DMEM) and the droplets were formed using an emulsification system. The study of the correlation between miRNA‐21 and cancer metastasis led to a better understanding of how miRNAs affect cellular functions at the single‐cell level.^[^ ^60^ ^]^ Brouzes and co‐workers used droplet microfluidics technology to develop a rapid and effective screening method for assessing cell viability and cytotoxicity. Cells were suspended in RPMI‐1640 prior to the droplet formation. After the live/dead percentage of cells was evaluated over 4 days of culture within droplets, the cytotoxicity screens proceeded (metabolic activity study), evaluating the culture with the cytotoxic agent for 24 h. Thus, this particular method not only allows precise evaluation of cell survival within the droplets but also enables cytotoxic screenings. This platform was demonstrated using monocytic U937 cells, showing a high degree of specificity and sensitivity. Finally, the authors state that one of the drawbacks is due to the conditioned cell proliferation within the droplets. This is attributed to the fact that only cells already committed to cell division before encapsulation can undergo further division. Conversely, other cells sense the confined droplet volume as a densely populated environment, inhibiting growth. The encapsulated milieu captures autocrine growth factors or metabolites, which contribute to this restricted growth environment.^[^ ^61^ ^]^

Beyond cell analysis, droplet microfluidics offers a unique vantage point for studying population heterogeneity. Observations from encapsulating individual *Saccharomyces cerevisiae* in microdroplets unveiled distinct growth rates within the same experimental conditions. These data highlight a possible existence of a subpopulation within the *S. cerevisiae* CEN.PK113‐7D cells, which would be impossible to observe under traditional analysis methods.^[^ ^62^ ^]^ Following the direction of nonmammalian cells, a new method of testing antibiotics’ susceptibility was developed using droplet microfluidics (Figure 2B). This method could test 32 antibiotics in the same machine while maintaining efficacy. Briefly, a suspension solution of *Escherichia coli* and the interest antibiotics with Mueller Hinton (MH) culture broth passed through an emulsification procedure, which originated picodroplets containing the previous suspension. Following the protocol, the growth and viability of the bacteria were assessed after an initial incubation period of 90 min. Subsequently, an additional 2 min were allotted for each successive antibiotic condition. This approach stands in contrast to conventional antibiograms, which typically necessitate a much longer incubation period of up to 48 h. The current main disadvantage of the technology is related to pathogen identification; hence, it is needed to pair it with a pathogen identification platform.^[^ ^63^ ^]^ In another study, droplet microfluidics was also applied, however, to encapsulate *microalgae*. *Microalgae* are a potential future source of biofuels, and promoting their fast growth is extremely relevant because it would increase biofuel production. Therefore, this work proposed to apply droplet‐based microfluidic for high‐throughput screening of single microalgae as it can evaluate growth and oil production at the single‐cell level. The potential of the platform was evaluated using a unicellular microalgae *Chlamydomonas reinhardtii*, which was suspended in Tris‐acetate‐phosphate (TAP) medium, and a colonial microalgae *Botryococcus braunii*, which was suspended in the Chu 13 medium. The platform successfully analyzed and compared *C. reinhardtii* growth and oil accumulation under various culture conditions, demonstrating its potential for efficient screening and analysis of numerous microalgal strains in a cost‐effective and time‐efficient manner. This system was anticipated to serve as a robust tool for high‐throughput investigation and screening of microalgal strains, offering significant benefits in terms of cost and time savings. The current problem with the strategy is due to the cytotoxicity of Nile red and dimethyl sulfoxide staining used for the analysis.^[^ ^64^ ^]^

Moreover, droplet microfluidics also has been applied in gene therapy studies. Specifically, the work of Xuan Li and colleagues aimed to develop an efficient method for lipofection for nonadhesive cell lines because the current method, lipoplex (cationic lipid–nucleic acid complex)‐mediated intracellular delivery, only had a 5% efficiency (Figure 2C). Thus, in this new approach, cells suspended in Opti‐MEM were coencapsulated with the plasmid, and the lipofectin (consisting of *N*‐(1‐(2,3‐dioleyloxy)‐propyl)‐*n*,*n*,*n*‐trimethylammonium chloride, and helper lipid dioleoyl–phophotidylethanolamine), which promotes the transfection. Importantly, this method significantly enhanced the plasmid's delivery efficiency from around ≈5% to approximately ≈50% across diverse suspension cell lines, such as K562, THP‐1, and Jurkat. This enhancement was accompanied by a concurrent decrease in cell‐to‐cell variability compared to traditional bulk methods. Additionally, successful implementation of this platform in CRISPR/CAS9‐mediated gene editing was demonstrated, effectively achieving a targeted TP53BP1 gene knockout in K562 cells. An ongoing pursuit involves the in vivo minimization of immune responses, upon the delivery of single‐cell‐encapsulating droplets post‐transfection.^[^ ^65^ ^]^

While the majority of microfluidic approaches involve oil and surfactants, other greener alternatives are being pursued. Aqueous two‐phase systems (ATPS) have been employed to create single‐cell units without the need for oil‐based solutions. ATPS is a biocompatible alternative to typical liquid–liquid extraction techniques that frequently use volatile organic solvents.^[^ ^66^ ^]^ ATPS solutions contain a mixture of two hydrophilic components that are incompatible with one another, such as two polymers or a polymer and a salt. Two immiscible aqueous phases may form in the systems depending on the component concentration.^[^ ^67^, ^68^ ^]^ Recently, Navi et al.^[^ ^69^ ^]^ applied the ATPS for single‐cell analysis, using dextran (where cells were mixed) and PEG as immiscible phases, combined with a partitioned ferrofluid, enabling paramagnetic manipulation of droplets. Although the successful generation of single‐cell droplets without the need for an oil‐based solution, these droplets suffer coalescence, only maintaining their stability for a maximum of 6 h. Wu et al.^[^ ^53^ ^]^ developed also an all‐aqueous approach to creating small droplets suitable for single‐cell analysis. For that, cells were resuspended in Isoton II or PBS buffer due to their high conductivity, and droplets were generated using capillary tubing and electrohydrodynamic actuation, integrated with microfluidic chips. This technology is a variant of its previously developed methodology (water‐in‐oil emulsion), using an electric field to generate droplets. However, similar to Navi's system, this technology exhibits limitations, such as decreased droplet stability and cell protection.

Overall, the current work on single‐cell droplets is very focused on cell analysis, encompassing fields like cellular compound capture, antibiotic susceptibility, heterogenetic studies, and genetic therapies. However, the scope of applications of single‐cell units generated via droplet‐based encapsulation is broadening, extending to domains such as T‐cell therapies and advanced cell sorting techniques (Table 1).^[^ ^70^, ^71^, ^72^, ^73^ ^]^ In fact, droplet microfluidics are currently being used for all those applications mainly due to their rapid, easy, and reproducible procedure. These droplets ensure direct cytocompatibility with suspension cell lines due to their liquid core, which also permits the flow of nutrients and metabolic by‐products while trapping molecules of interest inside the droplet. Nevertheless, their drawbacks are related to their medium/long‐term stability because they suffer coalescence and rapid liquid evaporation, and are associated with their difficult manipulation. Thus, cultures, analyses, or experiments longer than 6 h are challenging to fulfill. All in all, droplet microfluidics has a lot of potential, being very versatile, although it is not compatible with medium/long‐term assays.

### 3.2 Hydrogel‐Based Encapsulation

**Figure 3:**
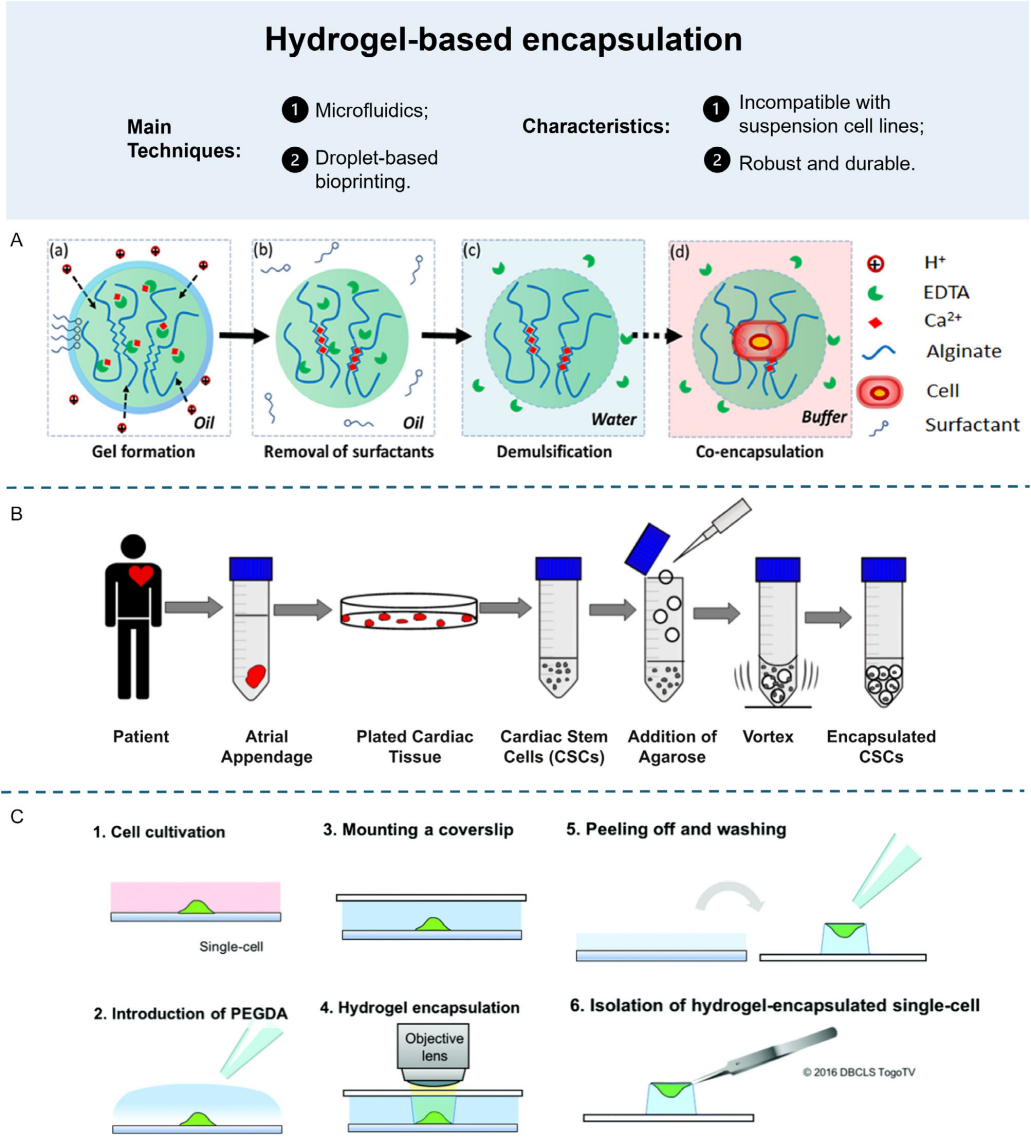
Main production techniques and characteristics of hydrogel‐based encapsulation. A) Single‐cell alginate microgels were produced to act as injectable bone fillers, utilizing MSCs to improve bone regeneration. Reproduced with permission.^[^ ^81^ ^]^ Copyright 2020, Elsevier. B) Single‐cell agarose microgels were fabricated directly incorporating patient cardiac stem cells individually, to improve acute retention. Reproduced with permission.^[^ ^75^ ^]^ Copyright 2013, Elsevier. C) Technique developed for genetic and morphological characterization of single cells. Single cells were first organized on a microfilter device based on their size and flexibility. Afterward, these cells were placed in PEGDA droplets, which are photopolymerizable, being ready for analysis. Reproduced with permission.^[^ ^74^ ^]^ Copyright 2019, The Royal Society of Chemistry.

Besides droplets, microfluidics can also generate microgels by adding a hydrogel precursor to the cell suspension. Although the majority use microfluidics as the main technique to form microgels, there are other methodologies, such as manual drop‐wise encapsulation,^[^ ^74^, ^75^, ^76^ ^]^ acoustic systems,^[^ ^77^, ^78^ ^]^ jet printing,^[^ ^79^ ^]^ hydroelectric atomization,^[^ ^36^ ^]^ among others (**Figure** **3**). This approach is usually preferred over the previous method, due to microgel robustness and durability, being more suitable for disease modeling or tissue engineering purposes. The process of gelation from droplet to microgel typically involves techniques such as ultraviolet radiation, temperature, or ionic cross‐linking.^[^ ^80^ ^]^ These microgels have been produced for a variety of applications, from cell analysis to tissue regeneration, or even cell therapies, demonstrating greater versatility than droplets.

Within the scope of tissue regeneration, single‐cell microgels have garnered particular attention in the context of bone therapies.^[^ ^36^, ^45^, ^81^, ^82^ ^]^ One of those examples is the work of Chuanfeng An and co‐workers, where single‐cell alginate microgels were produced to act as injectable bone fillers (Figure 3A). The single‐cell technique was chosen due to the advantages it presents when compared to multicellular strategies, mainly related to the microgel's easy delivery (enables direct intravenous delivery), and efficient nutrient and waste exchange for encapsulated cells. Furthermore, the incorporation of single cells allows the monitoring and analysis process into individual cell behavior and responses. Finally, the successful studies involving MSCs encapsulated in alginate microgels have demonstrated potential for extended cell viability and therapeutic effects post‐transplantation due to reduced immune clearance. Hence, MSCs were cultured individually in alginate microgels for 28 days in vitro, where the DNA content, the alkaline phosphatase activity, and the mineralization were evaluated periodically. The findings suggest that encapsulated MSCs suffer an accelerated osteogenic differentiation, with extended mineralization ability. Due to the results obtained in vitro, an in vivo experiment was conducted. This microgel‐based filler was applied in a rat tibial bone marrow ablation model to analyze its regeneration potential, which promoted a significant improvement in bone formation.^[^ ^81^ ^]^ Besides bone regeneration, single‐cell microgels have also shown promise in cartilage repair. Fredrikson et al. encapsulated single human primary chondrocytes in alginate microgels (Figure 3B). The authors used single cells because in vivo each chondrocyte is closely encapsulated in a pericellular matrix (PCM), which directly applies stimuli for chondrocyte mechanotransduction while protecting them from mechanical loads. Thus, the authors developed a method to better mimic the in vivo physiology in in vitro models. The data indicate that these microgels foster the development of collagen VI‐rich PCM surrounding single cells, and matrix synthesis when compared with monolayers.^[^ ^83^ ^]^ Expanding into cardiac regeneration, agarose microgels were fabricated directly incorporating patient cardiac stem cells. The hypothesis was that the encapsulation of stem cells would improve their acute retention, preventing mechanical clearance from the heart while protecting against anoikis. In fact, the direct application of microgels upon a myocardial infarction greatly boosted the rate of cardiac regeneration and enhanced engraftment and long‐term retention, maintaining it for 28 days.^[^ ^75^ ^]^

Apart from tissue regeneration, single‐cell microgels have proven invaluable in the domains of cell therapy and cell analysis. Zhichao Guan and co‐workers developed a platform to study the heterogeneity of cell populations by encapsulating cells in collagen microgels. When compared to more conventional techniques, this approach addressed challenges such as random cellular migration, nutrition depletion, and complex fluid shear while replicating the native 3D in vivo environment. This strategy enables long‐term single‐cell culture for precise analysis and drug screening.^[^ ^84^ ^]^

Droplet‐based bioprinting (DBB) has also been explored for single‐cell microgel formation. Evolving from microfluidics, DBB overcomes challenges associated with microfluidic‐based methods, such as the precise placement of cells inside the microdroplets, increasing the location accuracy and the reliability of the method.^[^ ^77^, ^85^, ^86^ ^]^ Moreover, DBB also allows for reaching printing rates of thousands of cells per second,^[^ ^77^ ^]^ making it suitable for large‐scale applications. Inkjet bioprinting, which is one type of DBB, has been used for single‐cell encapsulation among different applications, such as cell analysis^[^ ^87^ ^]^ and drug screening,^[^ ^85^ ^]^ among others. Interestingly, Liu et al. developed a dual‐nozzle cell printing system to fabricate intricate tumor models, leveraging both extrusion printing and alternating viscous and inertial force jetting (AVIFJ) techniques. The models were created by printing single‐cell laden microgels (gelatin–alginate gels) containing human umbilical vein endothelial cells (HUVECs) or HeLa cells and printing HepG2 spheroids. This system ensured precise droplet placement around cells/spheroids within the hydrogel, enabling the creation of complex heterogeneous tumor models with cellular interactions occurring within 100 μm of distance. Remarkably, the printed heterogeneous tumor models exhibited continuous cell viability, robust growth, and heightened cellular functions over 7 days. With its ability for single‐cell manipulation, easy nozzle switching, and image‐guided printing, this integrated cell printing system holds substantial promise for propelling the advancement of various vascularized tissue constructs in the near future. These intricate tissue models, characterized by microenvironments closely resembling in vivo conditions, diverse cell compositions, and intricate interactions, hold vast potential for diverse biological and pathological studies, particularly when paired with high‐throughput detection techniques like single‐cell sequencing.^[^ ^86^ ^]^ In another study, a customized alternating current (AC)‐pulse‐modulated electrohydrodynamic jet 3D printing system exhibited enhanced cytocompatibility, efficiency, and precision for encapsulating single cell into hydrogel microspheres, being a mixture of gelatin, hyaluronic acid, glycerol, and alginate. The results show high maintenance of cellular viability in the different cell types used (NIH 3T3 fibroblasts and HCT 116 colon carcinoma cell lines), and a great bioprinting capacity, being able to print different microsphere sizes (100–600 μm). These microspheres can be applied for cancer biology and drug screening, or even for in vitro multicellular spheroid model generation.^[^ ^79^ ^]^

In a completely different approach, Negishi and co‐workers developed a “gel‐based cell manipulation” method to separate adherent cells from culture dishes, overcoming the lack of strategies for analyzing single‐adherent cells (Figure 3C). Briefly, single cells are first organized on a microfilter device known as a microcavity array (MCA), based on their size and flexibility. Afterward, these cells were placed in PEGDA droplets, which are photopolymerizable using a fluorescent microscope. The specific cell was exposed to 405 nm light, causing the surrounding PEGDA to solidify into a hydrogel, which was around 300 μm in diameter, being handled manually. This technique was tested using three different cell lines (HeLa, A549, and NCI‐H1975), and resulted in efficient isolation (HeLa‐GFP − 95.6 ± 3.9%, A549 − 95.6 ± 3.9%, and NCI‐H1975 − 88.9 ± 9.6%), enabling genetic and morphological characterization.^[^ ^74^ ^]^

In conclusion, hydrogel‐based encapsulation proves to be a very versatile approach to a variety of applications. **Table** **2** offers an extensive overview of the techniques employed for the generation of single‐cell units through hydrogel‐based encapsulation. The table encompasses details, including polymer choice, cell type, and corresponding applications. Hydrogel‐based encapsulation strategies have gained significant prominence, particularly in the context of tissue model development and drug screening. This preference could be attributed to the enduring stability for long periods, versatility of the mechanical properties of microgels, and over time and their seamless compatibility with adherent cell cultures. Their compact size further allows for swift and noninvasive delivery, eliminating the need for surgical procedures. However, it is crucial to acknowledge that while these microgels demonstrate improved diffusion rates compared to larger hydrogels, they still exhibit limitations in nutrient and by‐product exchange when compared to droplets.

**Table 2:** Overview of works involving hydrogel‐based single‐cell encapsulation. MSC (mesenchymal‐derived stromal cell); EBV (Epstein Barr virus); HPC (hematopoietic stem cells); mESC (mouse embryonic stem cells); AML‐12 (alpha mouse liver 12); PEG (polyethylene glycol); CSC (cardiac stem cells); PEGDA (poly(ethylene glycol) diacrylate); DTT (dithiothreitol); *S. cerevisiae* (*Saccharomyces cerevisiae*); LAP (lithium phenyl‐2,4,6‐trimethylbenzoylphosphinate); *E. coli* (*Escherichia coli*); AC (alternating current); HUVECs (human umbilical vein endothelial cells); AVIFJ (alternating viscous and inertial force jetting); and GelMA (gelatin methacryloyl)

Author	Technique	Polymer	Cell Type	Application
Chuanfeng An et al.^[^ ^81^ ^]^	Emulsification (water–oil)	Alginate	MSC	Bone regeneration
Peter J. Attayek et al.^[^ ^149^ ^]^	Array‐based platform	Gelatin	K562 and EBV infected lymphoblastoid cells	Cell analysis
Jacob P. Fredrikson et al.^[^ ^83^ ^]^	Emulsification (water–oil)	Alginate	HPC	Cartilage regeneration
Zhichao Guan et al.^[^ ^84^ ^]^	Microcollagen gel array	Collagen	Kato III	Cell analysis
Tom Kamperman et al.^[^ ^51^ ^]^	Emulsification (water–oil)	Tyramine	MSC	Encapsulation efficiency
Tom Kamperman et al.^[^ ^10^ ^]^	Emulsification (water–oil)	Alginate and GelMA	Chondrocytes and MSC	Modular tissue engineering
Golnaz Karoubi et al.^[^ ^76^ ^]^	Manual drop‐wise encapsulation	Agarose	MSC	Cell analysis
Utkan Demirci et al.^[^ ^77^ ^]^	Acoustic system	Agarose	mESC, fibroblasts, AML‐12, Raji and HL‐1	Cell analysis, bioprinting
Hans Kleine‐Brüggeney et al.^[^ ^150^ ^]^	Emulsification (water–oil)	Agarose	MDA‐MB‐231, MDA‐MB‐468, and MCF7	Biomedical applications
Do Hyun Lee et al.^[^ ^151^ ^]^	Emulsification (water–oil)	Alginate	MCF–7, HepG2, U937 and *Chlorella vulgaris*	Single‐cell encapsulation enhancement
Rui Li et al.^[^ ^78^ ^]^	Acoustic system	Alginate	MCF‐7	Cell analysis
Qing Quan Liao et al.^[^ ^152^ ^]^	Emulsification (water–oil)	Alginate	MCF–7	Cell analysis
Philipp S. Lienemann et al.^[^ ^52^ ^]^	Emulsification (water–oil)	PEG	MSC	Cell analysis
Dongguo Lin et al.^[^ ^153^ ^]^	Emulsification (water–oil)	Alginate	HCT116 and HT29	Cell analysis
Audrey E. Mayfield et al.^[^ ^75^ ^]^	Manual drop‐wise encapsulation	Agarose	CSC	Cardiac therapy
Ryo Negishi et al.^[^ ^74^ ^]^	Manual drop‐wise encapsulation	PEGDA	HeLa, A549 and NCI‐H1975	Cell analysis
Simon Ng et al.^[^ ^154^ ^]^	Emulsification (water–oil)	PEG‐maleimide and DTT	*S. cerevisiae*	Enhancement of cell survival
Sander Oldenhof et al.^[^ ^155^ ^]^	Imaging‐assisted selective capture of cells	Dextran and LAP	NIH3T3 and A549	Cell isolation
Zehao Pan et al.^[^ ^36^ ^]^	Hydroelectric atomization	Alginate	MDA‐MB‐231 and MSCs	Bone regeneration
Kyun Joo Park et al.^[^ ^156^ ^]^	Emulsification (water–oil)	PEGDA	*E. Coli*	Platform for genetically modified microorganisms for practical applications
Fei Shao et al.^[^ ^45^ ^]^	Emulsification (water–oil)	Alginate	MSC	Bone regeneration
Yishen Tian et al.^[^ ^40^ ^]^	Emulsification (water–water)	Alginate	B16	T cell therapy
Bart M. Tiemeijer et al.^[^ ^22^ ^]^	Emulsification (water–oil)	Agarose	CD8 + T‐cell	T cell therapy
Yingfei Wang et al.^[^ ^20^ ^]^	Emulsification (water–water)	PEG	HepG2, HCCLM3, MHCC97L, and HHL‐5	Cell analysis
Jian Wang et al.^[^ ^79^ ^]^	AC‐pulse‐modulated E‐jet 3D printing system	Gelatin, hyaluronic acid, glycerol and alginate	NIH3T3 and HCT116	Tumor spheroids production
Yujie Zhang et al.^[^ ^82^ ^]^	Emulsification (water–water)	Alginate	MSC	Bone regeneration
Haoyue Zhang et al.^[^ ^157^ ^]^	Emulsification (water–water)	Alginate	MSC	Cell encapsulation platform
Liyuan Zhang et al.^[^ ^158^ ^]^	Emulsification (water–water)	Alginate	NIH3T3, HUVECs and MSC	Cell encapsulation platform
Tian kun Liu et al.^[^ ^86^ ^]^	Integration of extrusion printing and AVIFJ	Alginate	HeLa, HepG2, and HUVECs	Construction of artificial tissues
Venkatesh Selvam et al.^[^ ^159^ ^]^	Automated photopolymerization system	GelMA	HeLa	Cell analysis
Dan Liu et al.^[^ ^160^ ^]^	Emulsification (water–oil)	Alginate	MCF‐7	Cell encapsulation platform
Chuanfeng An et al.^[^ ^161^ ^]^	Emulsification (water–oil)	GelMA, methacrylated hyaluronic acid, nitrobenzene‐functionalized hyaluronic acid	MSC	Bone regeneration

In summary, hydrogel‐based encapsulation offers a versatile range of methodologies that present great promise. Yet, their incompatibility with suspension cells should be noted, urging a careful selection based on the specific cell type and intended application.

### 3.3 Nanoencapsulation

Inspired by the natural sporulation process observed in certain plant species, a new class of cellular hybrid systems emerged through a chemical strategy of single‐cell nanoencapsulation, resulting in what is referred to as “artificial spores.” This approach involves individually enclosing cells inside a thin yet robust shell, establishing a 3D “cell‐in‐shell” structure.^[^ ^88^ ^]^ The aim is to surround cells within biomimetic synthetic shells, ensuring protection from potential damage while facilitating the exchange of essential nutrients and waste products.^[^ ^89^, ^90^, ^91^ ^]^ The difference between nanoencapsulation and other conventional surface‐functionalized cells relies on their ultrathin shell (100 nm), robustness, selective permeability, degradability, and functionality.^[^ ^92^, ^93^ ^]^ The artificial shells can withstand mechanical stresses, arising from osmotic pressures and dehydration, preserving their original shape under damaging conditions. Additionally, the artificial shell's mechanical toughness can control cell growth and division by partially withstanding the biological forces involved. The artificial shell's porosity can be chemically tailored to allow the passage of small molecules through the shell (in and out of the cytoplasm), but penetration by outside aggressors, such as lytic enzymes and macrophages, can be selectively blocked to maintain cell viability.^[^ ^94^ ^]^ Moreover, it is often required that the shell possesses the ability to disintegrate as needed in response to stimuli or through controlled degradation. Equally significant is the consideration of the shell's functionalization during or after construction for precise external recognition and interaction.^[^ ^95^ ^]^

**Figure 4:**
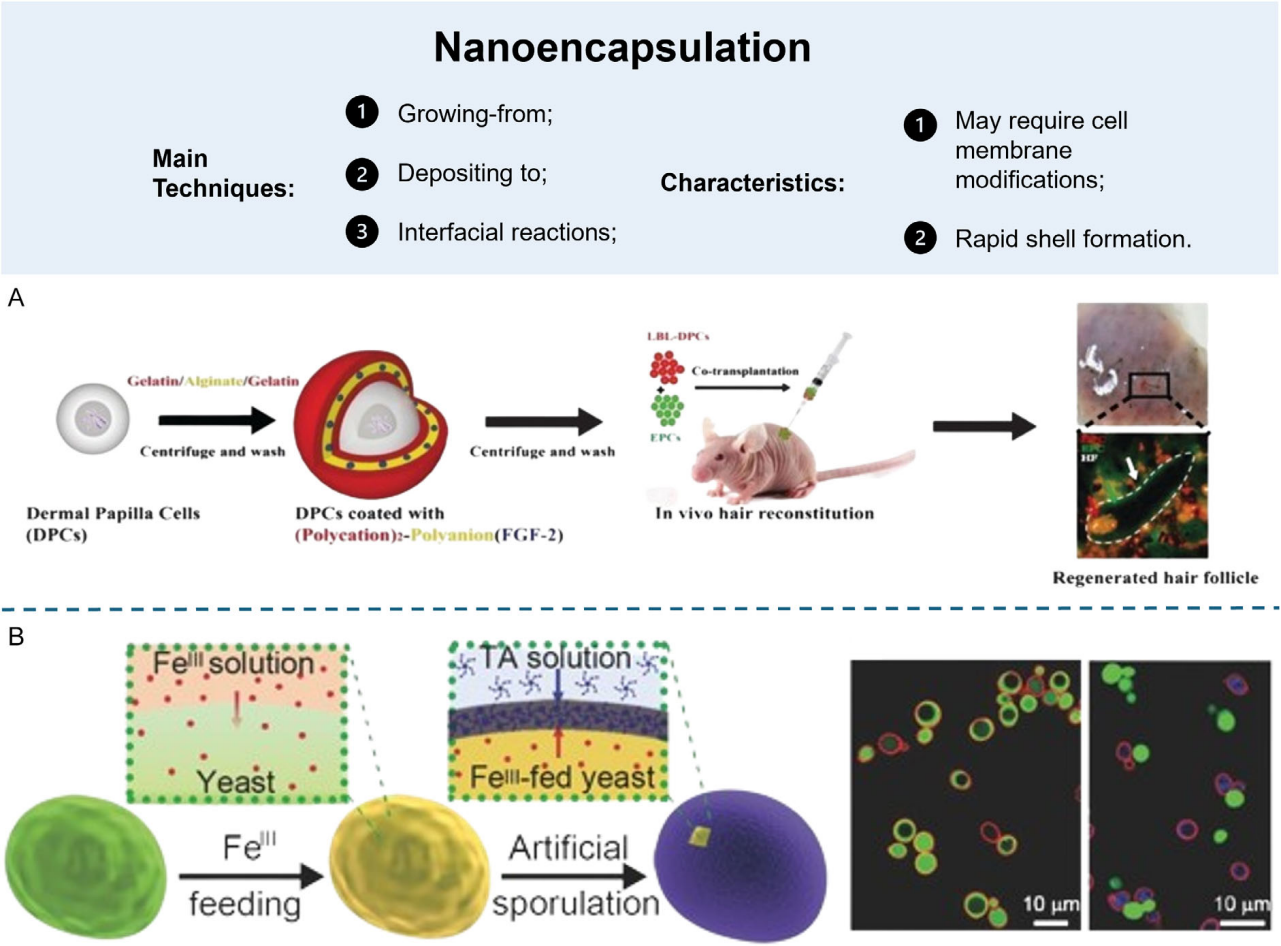
Main production techniques and characteristics of nanoencapsulation approaches. A) LbL was used to address hair loss problems, conjugating alginate with gelatin, to encapsulate dermal papilla cells (DPCs). Reproduced with permission.^[^ ^104^ ^]^ Copyright 2017, WILEY‐VCH Verlag GmbH & Co. KGaA, Weinheim. B) A cytocompatible technology able to form an autonomous shell around yeast cells, to isolate and protect living cells from harmful external environments. Cells were exposed to a TA solution after being pretreated with Fe(III). The shell was formed when Fe(III) entered in contact with TA, forming a metal–organic complex. Reproduced with permission.^[^ ^111^ ^]^ Copyright 2017, WILEY‐VCH Verlag GmbH & Co. KGaA, Weinheim.

Various methods used to create cells enclosed in shells can be categorized using different features (**Figure** **4**). For this conversation, the categorization was based on how the capsule forms, which can be split into three main groups: the depositing‐to approach, the growing‐from approach, and interfacial reactions.^[^ ^96^ ^]^ An alternative classification can be established based on shells’ degradability and their influence on biological function. Shells can be categorized as active or passive, where the classification of shells depends on whether their functions are directly tied to reaction‐related aspects (e.g., chemical or biological reactions), shells are designated as active when their roles involve such reactions. Conversely, shells are regarded as passive if their features can block the penetration of molecules or can recognize specific molecules, without direct reaction implications. Within those groups, shells can be also classified as either static (nondegradable) or dynamic (degradable).^[^ ^91^ ^]^

#### 3.3.1 Depositing‐to Approach

In this approach, materials such as metals, polyelectrolytes, or small molecules are directly deposited on cell surfaces through physicochemical interactions. For instance, in the case of the layer‐by‐layer (LbL) technique, the layer's thickness can be altered in each deposition period (varying the time or concentration of the polyelectrolyte), or it can be constant. Hence, the deposition procedure can be repeated to obtain the desired shell thickness.^[^ ^97^, ^98^ ^]^ The LbL technique can be strategically designed to impart degradability to the layers.^[^ ^99^ ^]^ For instance, Moon et al. developed layers containing alginate alternating with cationic starch, which is degraded by the action of α‐amylase,^[^ ^100^ ^]^ although there are other available strategies, such as through pH‐triggered degradation,^[^ ^101^ ^]^ near‐infrared light,^[^ ^102^ ^]^ redox‐activated,^[^ ^103^ ^]^ among others.

A very interesting application for single‐cell units using the LbL technology was developed to address hair loss (Figure 4A). Derma papilla cells (DPCs) were individually surrounded by a membrane containing three layers (gelatin–alginate–gelatin), with gelatin acting as the polycation and alginate as the polyanion. Afterward, the encapsulated single cells were treated with calcium to promote spheroid aggregation, closely mimicking the natural microenvironment of these DPCs. Spheroids viability was tested in vitro for 7 days and in vivo on C57BL/6 mice to assess the induction of hair growth. Results of spheroids implantation revealed a very visible hair growth, showing promise for applications in tissue engineering and regenerative medicine, particularly in the treatment of hair loss‐related diseases.^[^ ^104^ ^]^ In another approach, a quick and easy bottom‐up method known as the cell‐accumulation technique was created by coating single cells with fibronectin–gelatin nanofilms. A 6 nm nanofilm was obtained surrounding each cell surface after nine steps of immersion in the polymers. The cell‐accumulation technique was achieved because the nanofilms interact with the α5β1 integrin receptor of the cell membrane, which consequently promotes the cell–cell interaction, leading to cell accumulation. The viability of the method was confirmed on human dermal fibroblast cells (hFC), HUVECs, and HepG2, resulting in a high percentage of survival. The different types of cells were able to form 3D multilayered tissues, which, when embedded with HUVECs, could develop capillary networks with tubular morphologies.^[^ ^105^ ^]^ In an interesting depositing‐to approach, human adipose‐derived mesenchymal stromal cells (hASCs) were individually and partially coated with silica, where the coating acts as an individual adhesion spot for adherent cells. The method's essence lies in the creation of silica backpacks on individual hASCs through controlled electrostatic interactions with a specially tailored compound (chitosan–carnitine), followed by in situ generation of silica. Noteworthy is the fact that this process maintained cell viability without the need for heavily charged polycations. A remarkable outcome of this process is the mechanical reinforcement it imparts to cell membranes via the silica backpacks, thereby ensuring cell survival in suspension environments. This is a vital consideration for procedures such as inject‐based applications, 3D bioprinting, and microfluidics, where cell membrane integrity is essential. The mechanotransduction properties could be harnessed for also controlling cell differentiation into an osteogenic phenotype.^[^ ^106^ ^]^

#### 3.3.2 Growing‐From Approach

In the growing‐from approach, a precursor adheres to the cell surface, triggering the formation of a cell‐revesting capsule under an appropriate stimulus.^[^ ^96^ ^]^ Most of the investigation done on this strategy has been applied to microbial^[^ ^107^, ^108^ ^]^ or algae^[^ ^109^ ^]^ encapsulation. Nevertheless, this strategy has also been extended to mammalian cells.^[^ ^8^ ^]^ For instance, mouse MSCs have already been encapsulated using this type of technique, to obtain more control over the mechanical properties of the microgel, and consequently influence the biological functions of cells. Briefly, mouse MSCs and murine preadipocyte cells (OP9s) were exposed to a suspension of calcium carbonate nanoparticles, allowing concentration‐dependent passive adsorption onto the cell surface. Following the washing procedure, cells were mixed with alginate and subsequently with acetic acid. This sequence caused the release of calcium from nanoparticles and consequently, the cross‐linking of the polymer, resulting in the formation of a microgel. Single‐cell microgels were successfully intravenously injected into syngeneic mice after confirming cell viability postencapsulation. Results indicated that the encapsulation of MSCs delayed the clearance kinetics after implantation. Furthermore, single‐cell units were shown to be more sensitive to exogenous factors compared to multicell strategies.^[^ ^8^ ^]^ A revolutionary method was described by Niu and co‐workers, introducing the cytocompatible controlled radical polymerization (CRP) technique, to generate structurally defined synthetic polymers on living cell surfaces. Briefly, a chain‐transfer agent (2‐(butylthiocarbonothioyl) propionic acid, BTPA) was added to the cell surface by using covalent (yeast) or noncovalent (mammalian) insertion techniques. These added groups were then used as polymerization initiators to generate the capsule. The amount of polymer chains grafted on the cell surface was significantly higher compared to traditional polymer grafting strategies. The cytocompatibility was assessed on yeast cells, which retained their viability for 70 h, and on human Jurkat cells, demonstrating a 90% survival rate. This pioneering cytocompatible CRP technique holds potential for diverse applications, including rewriting signaling pathways or controlling spatial distributions of cell surface‐grafted polymers, as envisioned by the authors.^[^ ^110^ ^]^

#### 3.3.3 Interfacial Reactions

The interfacial reaction considers the theoretical capacity of cells to recognize dangerous molecules in their surroundings and release “catching” components to generate neutralized pericellular layers. This strategy holds significant promise, as the formation of the interfacial shell could theoretically offer long‐term preservation, defense against damaging attacks, and autonomous and self‐regulated mechanisms.^[^ ^96^ ^]^

One strategy for mimicking this shielding technique of certain living cells is through biphasic interfacial supramolecular self‐assembly, which implies contact‐based self‐assembly between two immiscible phases, usually oil and water, each containing a reactive component (Figure 4B). For instance, Kim et al. developed a cytocompatible technology able to form an autonomous shell around yeast cells, to isolate and protect living cells from harmful external environments. In detail, cells were exposed to a tannic acid (TA) solution after being pretreated with Fe(III). Fe(III)–TA metal–organic complex (Fe(III)–TA–MOC) shells were formed when the passive efflux of Fe(III) made contact with TA in the pericellular space. This finding raises the prospect of creating active techniques in which cells serve as reservoirs for the release of chemicals that instantly interact with other elements of the extracellular space to create stable and uniform nanocoatings.^[^ ^111^ ^]^ In another study, it was developed a hydrogel shell with a liquid core with the potential to be used in a variety of applications, as well as single‐cell encapsulation, although the authors did not test the cytocompatibility of the technology. Briefly, the approach leveraged an ATPS within consistently sized aqueous droplets generated through microfluidic emulsification. These droplets contained dextran and two types of tetra‐arm poly(ethylene glycol) (tetra‐PEG) macromonomers, producing ATPS droplets with a dextran‐enriched core and a tetra‐PEG macromonomer‐rich shell. Furthermore, a subsequent autonomous cross‐linking of tetra‐PEG macromonomers within the shell fabricates the hydrogel microcapsules, eliminating the need for external cross‐linking triggers. The resultant microcapsules are a robust tetra‐PEG hydrogel shell known for its durability. This technique holds promise within biomedical fields like drug delivery, bioreactors, and regenerative medicine, offering the potential for modifying hydrogel microparticle structure by adjusting the tetra‐PEG macromonomer reaction rate.^[^ ^112^ ^]^

Overall, nanoencapsulation emerges as a versatile category for single‐cell encapsulation, allowing the rapid and efficient formation of microgels or capsules around individual cells. **Table** **3** showcases the existing research work in the realm of nanoencapsulation. In conclusion, the concept of nanoencapsulation has introduced a groundbreaking shift, manifesting as “artificial spores,” encapsulating individual cells within robust shells. This innovative approach establishes a distinctive 3D “cell‐in‐shell” structure, yielding a range of benefits. The ultrathin yet mechanically resilient shell ensures protection against mechanical stress and external factors while permitting controlled nutrient exchange. Selective degradation response offers tailored release mechanisms, further enriched by shell functionalization for precise interactions, paving the way for versatile applications across biomedicine. Nevertheless, challenges are inherent, mainly due to balancing shell durability and controlled degradation. Precisely tuning shell permeability for molecule exchange while maintaining cellular integrity presents intricate engineering demands. The nanoencapsulation process necessitates meticulous reaction control for consistent outcomes. The exploration of active versus passive shells adds complexity, requiring a thorough grasp of their impact on cellular responses. Overall, the trajectory of nanoencapsulation points to an exciting future, ensuring easy and rapid techniques for autonomous shielding.

**Table 3:** Overview of works involving nanoencapsulation of single cells. LbL (layer‐by‐layer); PAH (poly(allylamine hydrochloride)); PSS (poly(styrenesulfonate sodium salt)); *S. cerevisiae* (*Saccharomyces cerevisiae*); PMAA (poly(methacrylic acid); NSC (neural stem cells); EPC (endothelial progenitor cell); *C. reinhardtii* (*Chlamydomonas reinhardtii*); ASC (adipose‐derived stromal cell); PEI (poly(ethyleneimine)); PDPA (poly(2‐diisopropylaminoethyl methacrylate)); PLL (ε‐poly‐l‐lysine); iPSC (induced pluripotent stem cells); FC (dermal ﬁbroblast cells); NPC (neural progenitor cells); Ac4ManNAz (*N*‐azidoacetylmannosamine‐tetraacylated); MSC (mesenchymal‐derived stromal cell), PEGA‐1k (methoxy‐PEG acrylamide‐1k), BTPA (2‐(butylthiocarbonothioyl) propionic acid); *E. coli* (*Escherichia coli*); *L. acidophilus* (*Lactobacillus acidophilus*); RBC (red blood cells);TN‐PEG (tetra(succinimidyloxyglutaryl) polyoxyethylene); and TA‐PEG (tetra (aminopropyl)polyoxyethylene)

Author	Technique	Material	Cell type	Structure formed	Application
Alberto Diaspro et al.^[^ ^108^ ^]^	Depositing‐to (LbL)	PAH and PSS	*S. cerevisiae*	Shell	Cell analysis
Irina Drachuk et al.^[^ ^94^ ^]^		PMAA	*S. cerevisiae*	Shell	Biosensors
Sang Yeong Han et al.^[^ ^162^ ^]^		Eggshell membrane hydrolysates and coffee melanoidin	*S. cerevisiae*	Shell	Drug delivery, tissue engineering, and nanobiomedicine
Wenyan Li et al.^[^ ^163^ ^]^		Gelatin and alginate	NSC	Shell	Cell analysis
Bo Jie Lin et al.^[^ ^164^ ^]^		Gelatin and alginate	EPC	Shell	Hair follicle regeneration
Nikolaj Kofoed Mandsberg et al.^[^ ^109^ ^]^		Tannic acid and iron (III)	*C. reinhardtii*	Shell	Cell survival enhancement
Hee Chul Moon et al.^[^ ^100^ ^]^		Cationic starch and alginate	*S. cerevisiae*	Shell	Degradable capsule
Ji Hun Park et al.^[^ ^107^ ^]^		Tannic acid and iron (III)	*S. cerevisiae*	Shell	Cell encapsulation platform
Jin Wang et al.^[^ ^104^ ^]^		Gelatin and alginate	DPC	Shell	Hair follicle regeneration
Marta Maciel et al.^[^ ^106^ ^]^		Silica and chitosan	ASC	Shell	Cell partial encapsulation
Sung Ho Yang et al.^[^ ^95^ ^]^		PEI and PSS	*S. cerevisiae*	Shell	Cell encapsulation platform
Kang Liang et al.^[^ ^101^ ^]^		PDPA and PMAA	JAWS II	Shell	Degradable capsule
Alisa L. Becker et al.^[^ ^103^ ^]^		PMAA and PLL	PC‐3	Shell	Degradable capsule
Akihiro Nishiguchi et al.^[^ ^105^ ^]^		Fibronectin and gelatin	FC, HUVECS, and HepG2	Shell	Tissue engineering and drug screening
Mengzhen Han et al.^[^ ^165^ ^]^		Chitosan and liposomes	*E. coli*	Shell	Colitis‐related diseases therapy
Luis P. B. Guerzoni et al.^[^ ^166^ ^]^		Fibronectin and gelatin	iPSC	Shell	Cardiac therapy
Yanyun Fang et al.^[^ ^41^ ^]^	Growing‐from	DNA‐copolymer	T cell, Jurkat, and CD19 CAR‐T	Shell	T cell therapy
Byeongtaek Oh et al.^[^ ^167^ ^]^		Ac4ManNAz	NPC	Shell	Cell therapy
Jianmin Yang et al.^[^ ^168^ ^]^		Acrylamide	HeLa and MSC	Shell	Cell encapsulation platform
Jia Niu et al.^[^ ^110^ ^]^		PEGA‐1k, BTPA, Eosin Y, and triethanolamine	*S. cerevisiae* and Jurkat	Shell	Cell encapsulation platform
Angelo S. Mao et al.^[^ ^8^ ^]^		Alginate	MSC and OP	Microgel	Control of tissue assembly and cell delivery
Beom Jin Kim et al.^[^ ^169^ ^]^	Interfacial reactions	Tannic acid and ferric ions	*S. cerevisiae, E. coli*, *L. acidophilus*, Jurkat, and RBC	Shell	Modulation of the material structures and properties
Takaichi Watanabe et al.^[^ ^112^ ^]^		TN‐PEG and TA‐PEG	–	Hydrogel Shell with a liquid core	Cell encapsulation, biosensors, and drug delivery

## 4 Sorting Strategies

As previously discussed, the majority of single‐cell encapsulation methodologies present an important drawback related to the Poisson distribution, leading to an approximate 37% efficiency in individual cell encapsulation, while the remaining units are either empty or multicell. Moreover, in order to reduce the number of multicell units, low cellular concentrations are used. Consequently, it is necessary to have an effective sorting of empty units from the single‐ and multicell ones. Currently, there are different approaches capable of addressing this challenge, such as fluorescence‐activated cell sorting (FACS), size sorting, and magnetic‐based separation.

### 4.1 Common Strategies

**Figure 5:**
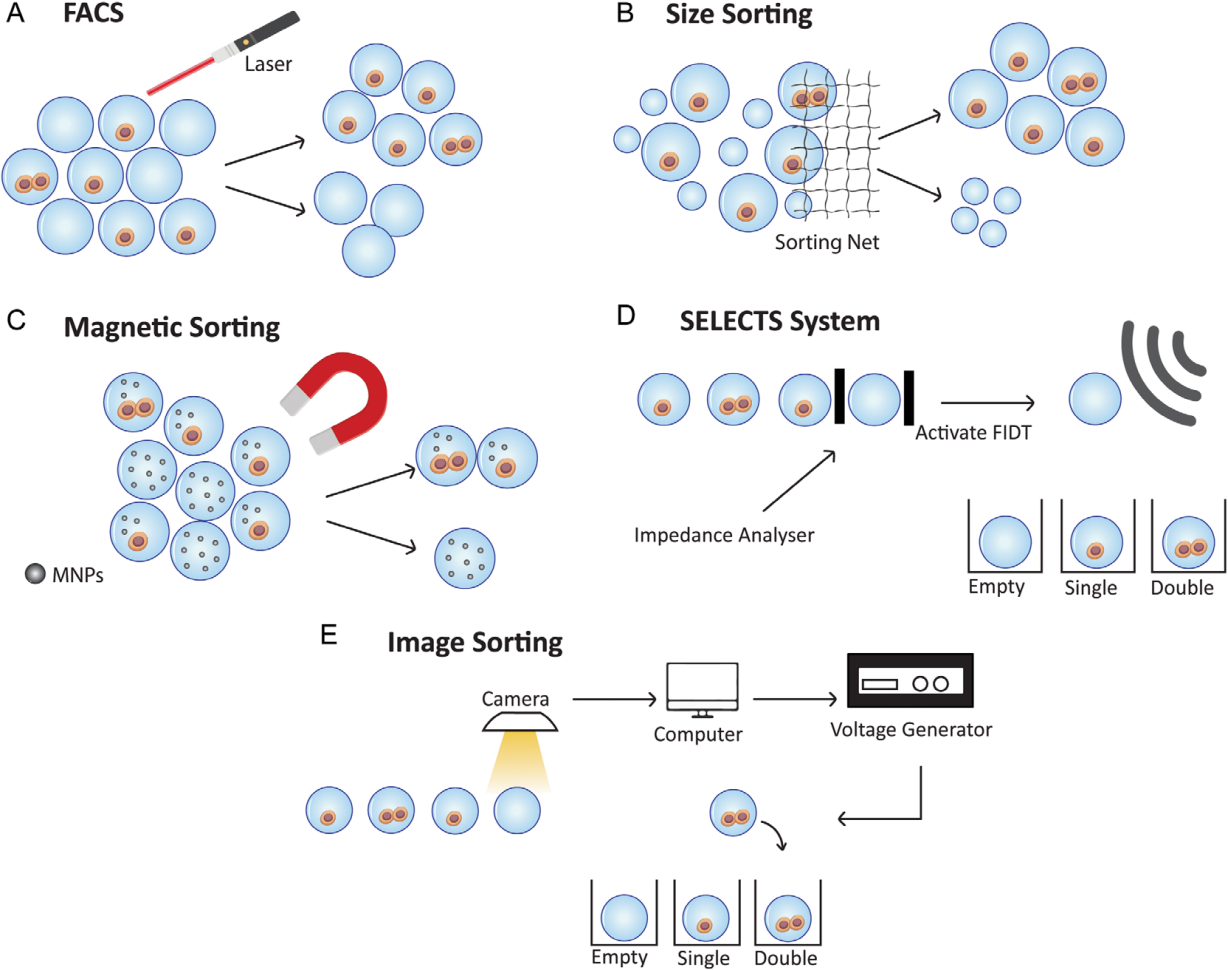
Sorting strategies to obtain single‐cell units. A) FACS discriminates single‐cell units from empty ones through fluorescence signals. B) Size‐based sorting separates single‐cell units from empty microgels, which tend to be smaller. C) The encapsulation of MNPs allows the sorting of single‐cell units through magnetic impulses. D) Acoustic field‐based sorting, following electrical screening, distinguishes single‐cell units from empty and multicell units. E) Artificial intelligence software facilitates the sorting of single‐cell units, distinguishing them from empty and multicell units.

FACS is an established technique for single‐cell unit sorting. Using the unique light scattering and fluorescence properties of each cell, it offers a way for dividing a heterogeneous mixture of living cells into two or more containers, one cell at a time. It is an efficient scientific tool that records fluorescence signals from individual cells quickly, objectively, and quantitatively while also physically separating cells of specific interest.^[^ ^113^ ^]^ In the specific context of encapsulation, FACS allows the separation of empty units from those with cells, utilizing the fluorescence emitted upon laser beam excitation for identification and subsequent separation (**Figure** **5**A). In fact, FACS is the main technique used to sort single‐cell units.^[^ ^8^, ^10^, ^19^, ^41^, ^62^, ^73^, ^74^, ^75^, ^76^, ^114^, ^115^ ^]^

Size sorting (Figure 5B) considers that the majority of units with cells present a larger size than empty units. Jing et al. applied this methodology in their droplets, implementing size‐based sorting within the deterministic lateral displacement micropillar channel. The critical dimension for separation was defined by a geometric design. Although empty units show a tendency to be smaller, the efficiency rate reaches around 80%.^[^ ^116^ ^]^

Different sorting approaches take advantage of the magnetic properties (Figure 5C) of some materials. Navi et al. illustrated a sorting method involving diamagnetic droplet separation using ATPS capsules. In their study, a ferrofluid was included in the PEG phase (phase II). As a result, upon droplet formation, the ferrofluid in the PEG phase was drawn to the magnet, repelling the droplets. The diamagnetic effect is increased inbiggerdroplets, which are the ones that containcells, while in the empty droplets the effect is attenuated. Thus, single‐cell droplets possess a larger diamagnetic effect, being repelled.^[^ ^69^ ^]^ However, this sorting strategy may have some limitations, such as observed by Kamalakshakurup and Lee,^[^ ^117^ ^]^ where single‐cell droplets were smaller than empty droplets, potentially affecting accurate separation. Jo et al.^[^ ^118^ ^]^ described a sorting methodology, where cells were encapsulated with magnetic nanoparticles (MNP) within droplets. The sorting was based on the principle that single‐cell droplets would contain fewer MNPs, leading to a decreased magnetic charge, while empty droplets display a greater magnetic response. In contrast to Navi's method, this one addresses size‐related challenges by correlating magnetic response with cell presence. However, this approach relies on uniform MNP distribution and predictable interactions, which can introduce complexities and uncertainties into the sorting process.

### 4.2 Highly Precise Strategies

Despite these sorting strategies are well‐studied and broadly used, they fail in effectively sorting single‐cell units from multicell ones. Therefore, in order to ensure the homogeneity of the final product, more refined and precise techniques are required. Zhong^[^ ^119^ ^]^ introduced the Selectable Label‐free Encapsulated Cell‐in‐droplet Sorting system (SELECTS system). The SELECTS system combines label‐free high‐throughput electrical screening for droplet content analysis, with a biocompatible acoustic sorting in a microfluidic chip for physical separation (Figure 5D). The method exhibits excellent specificity (98.9%), for distinguishing empty and cell‐encapsulated droplets and even rejecting multicell units (60%) due to the high impedance screening accuracy. The system obtained a final efficiency of 90%. Apart from the SELECTS system, another approach has been reported as capable of sorting encapsulation units by the number of cells. Imaging sorting systems (Figure 5E) comprise an excellent alternative, allowing the identification and separation of the encapsulation units by the number of cells, or even their morphology. This approach is based on a combination of hardware and software that includes cell detection and sorting algorithms. The hardware contains a droplet image recognition system, a microfluidic controller, and a microfluidic chip with liquid electrodes. The software is the command center, responsible for data interpretation and decision‐making. This alternative is capable of achieving a sorting efficiency superior of 90%.^[^ ^120^ ^]^

Overall, each strategy possesses its unique set of advantages and disadvantages. When prioritizing accessibility and ease of use, the most effective approach is to employ conventional sorting methodologies. This allows for moderate‐to‐good sorting efficiency, offering a quick and straightforward sorting process. On the other hand, if the goal is to achieve higher sorting efficiency, opting for highly precise sorting strategies is essential. Despite the requirement for more sophisticated infrastructure, these methods enable the sorting of large sample volumes within seconds to minutes. In summary, FACS stands out as the strategy that optimally combines all the benefits. It delivers efficient sorting in a rapid and user‐friendly manner, although requires the availability of specific equipment.

## 5 Conclusion and Future Perspectives

Single‐cell encapsulation has emerged as a potent and versatile technique with the potential to revolutionize several fields, ranging from regenerative medicine to drug delivery and cellular studies. It holds immense promise, underscored by the unique advantages it offers. The small size of the single‐cell units allows direct delivery into the blood system, enabling precise control over biodistribution within the body. Moreover, it presents better diffusion rates when compared to traditional encapsulation strategies, enabling control over cell proliferation, survival, and migration.^[^ ^7^, ^8^, ^10^, ^11^ ^]^ Nevertheless, there is not yet a licensed product applied in the field of tissue engineering and regenerative medicine, which can be explained not only by the novelty of the technology, but also due to the variability associated with the use of natural products and their limited production throughput. The utilization of droplet microfluidics, the development of hydrogel‐based encapsulation, and the creation of “artificial spores” through nanoencapsulation have each showcased distinct advantages and challenges.

Droplet microfluidics offers rapid and reproducible procedures, ensuring direct cytocompatibility with suspension cell lines. However their medium‐ to long‐term stability is challenged by coalescence and fast liquid evaporation. This highlights the need for further development in stability enhancement for extended applications. Similarly, hydrogel‐based encapsulation has gained prominence, particularly in tissue model development and drug screening. While these microgels provide improved stability and compatibility with adherent cell cultures, their effectiveness in nutrient exchange compared to droplet‐based methods remains a consideration. Despite this limitation, hydrogel‐based strategies hold promise, particularly when considering the selection of appropriate methodologies based on the specific cell type and intended application. The concept of nanoencapsulation offers a unique 3D “cell‐in‐shell” structure, affording benefits, such as mechanical protection against stressors and controlled nutrient exchange. Selective degradation responses provide tailored release mechanisms, while shell functionalization opens avenues for precise interactions, contributing to versatile biomedical applications.

Moreover, the production of single‐cell units frequently relies on microfluidic principles, with sorting methodologies representing a critical aspect. The efficiency of single‐cell unit generation is often limited, due to the Poisson distribution. To address this, the sorting of empty units from single‐ and multicell units becomes crucial. Existing strategies, such as FACS, size sorting, and magnetic‐based separation, have emerged to achieve this separation. However, these methods find challenges such as high costs for FACS, or limited versatility for the remaining approaches. In fact, artificial intelligence may be a great help in solving some of these challenges.^[^ ^120^ ^]^ Developing accessible, streamlined, and rapid sorting methodologies is imperative to maximize the efficiency of single‐cell unit production and the successful deployment of encapsulated cells for different applications.

The trajectory of single‐cell encapsulation promises a future marked by autonomy and precision. With an array of techniques offering unique strengths and acknowledging limitations, the field's evolution holds the potential for unprecedented breakthroughs. As the field progresses, refining sorting methods, developing more efficient encapsulation techniques, and exploring new applications will contribute to harnessing the full potential of single‐cell encapsulation, propelling it toward a future where personalized medicine, regenerative therapies, and cellular research find novel solutions and unique possibilities.

## Conflict of Interest

The authors declare no conflict of interest.
